# Natural Phytochemical and Visible Light at Different Wavelengths Show Synergistic Antibacterial Activity against *Staphylococcus aureus*

**DOI:** 10.3390/pharmaceutics16050612

**Published:** 2024-05-01

**Authors:** Jae-Young Jeong, You-Jin Hwang

**Affiliations:** 1 Department of Biohealth & Medical Engineering, College of IT Convergence, Gachon University, Seongnam 13120, Republic of Korea; jini656565@gachon.ac.kr; 2 Department of Biomedical Engineering, College of IT Convergence, Gachon University, Seongnam 13120, Republic of Korea

**Keywords:** photodynamic therapy, visible light, natural phytochemical, antibacterial activity, *Staphylococcus aureus*, synergistic effect

## Abstract

As the risk of antibiotic-resistant bacteria increases, interest in non-antibiotic treatment is also increasing. Among the methods used in non-antibiotic therapy, natural antibiotics such as essential oils have disadvantages such as low efficiency. In the case of phototherapy, the light used for antibacterial activities has low penetration into the human body because of its short wavelength, making it of low medical utility. To solve this problem, this study aimed to determine conditions for enhancing the antibacterial activity of natural phytochemicals and visible light. Four natural phytochemical extracts that showed high antibacterial properties in previous studies were analyzed. Synergistic effects on antibacterial activity and cytotoxicity were determined when natural phytochemical extracts and visible light were simultaneously used. As a result, it was confirmed that the antibacterial activity increased by four times when *Sanguisorba officinalis* L. was irradiated with 465 nm for 10 min and 520 nm for 40 min, and *Uncaria gambir* Roxb. was irradiated with 465 nm for 10 min and 520 nm for 60 min compared to when *Sanguisorba officinalis* L. and *Uncaria gambir* Roxb. were used alone. The synergistic effect on antibacterial activity was independent of the absorption peak of the natural phytochemical extracts. In addition, in the case of natural phytochemical extracts with improved antibacterial activity, it was confirmed that the improvement of antibacterial activity was increased in inverse proportion to the light irradiation wavelength and in proportion to the light irradiation time. The antibacterial activity was enhanced regardless of antibiotic resistance. In the case of cytotoxicity, it was confirmed that there was no toxicity to A549 cells when treated with 465 nm, the shortest wavelength among the natural phytochemical extracts. These results show how to replace blue light, which has been underutilized due to its low transmittance and cytotoxicity. They also demonstrate the high medical potential of using natural phytochemical and visible light as a combination therapy.

## 1. Introduction

*Staphylococcus aureus* is the most common and deadly pathogen involved in purulent acute bacterial skin and skin structure infections [1,2]. *S. aureus* can lead to various skin diseases such as folliculitis, impetigo, abscesses, furuncles, furunculosis, mastitis, and hidradenitis suppurativa by causing local and systemic infections and producing staphylococcal toxins through the skin [1,2,3]. More than 2.8 million antibiotic-resistant infections occur annually in the United States, accounting for 35,000 deaths [4]. In Korea, the estimated annual number of antibiotic-resistant infections in 10 hospitals in 2017 was 7979, resulting in about 3280 deaths and socioeconomic losses of USD 294,505,002 [5]. The use of empirical and broad-spectrum antibiotics to eliminate antibiotic-resistant bacteria creates a vicious cycle of generating other antibiotic-resistant bacteria [4]. Therefore, interest in non-antibiotic therapies that can replace antibiotics is increasing.

Methods emerging as non-antibiotic therapies include treatment using natural phytochemical extracts and light-based antibacterial therapy [6,7]. Representative natural phytochemical extracts used for antibacterial therapy include essential oils, which are volatile components made from aromatic plants. About 3000 essential oils are known, of which 300 are certified by the US Food and Drug Administration as generally safe for humans. They are widely used in cosmetics, perfumes, food preservatives, and additives [6,7]. Since these natural phytochemical extracts have low cost, high accessibility, structural diversity, various modes of activation, high biocompatibility, potential anti-biofilm properties, environmental friendliness, and low probability of antibiotic resistance, they are attracting attention as environmentally friendly non-antibiotic therapies [6,7,8,9].

Light-based antibacterial therapy, including antibacterial photodynamic therapy (aPDT) and ultraviolet C (UVC) irradiation therapy, is being studied as a method for treating local infection [10]. aPDT essentially requires a photosensitizer (PS), molecular oxygen, and light of a specific wavelength to activate the PS, and for this antibacterial activity to be active, the PS must possess a large amount of singlet oxygen quantum [11]. Regarding the action mechanism for the antibacterial activity of aPDT, it involves reactive oxygen species (ROS) by combining the PS with visible or near-infrared rays, causing cell death [11,12,13,14,15]. The antibacterial activity of UVC occurs by causing DNA damage through various mutagenesis and cytotoxic DNA lesions [16]. Light-based therapy has advantages such as fast antibacterial activity unrelated to antibiotic resistance, high treatment repeatability, fewer side effects, and high compatibility with other treatments [10,11,12,14,17]. However, in the case of aPDT, there are disadvantages in that a photosensitizer must be separately administered. In addition, it is difficult to have a bacteria-specific antibacterial activity [10,17]. On the other hand, since it can damage host cells, active research on human use has not been conducted in the case of ultraviolet C irradiation [10,16].

Blue light therapy, which appears to compensate for these disadvantages, is receiving a lot of attention because it has an antibacterial effect alone without needing additional photosensitizers. Its antibacterial mechanism remains unclear; a representative hypothesis is that the generation of ROS is induced by photoactivation of the endogenous photosensitive porphyrin possessed by bacteria [7,18,19,20]. Blue light is known to cause much less damage to host cells than ultraviolet light [10,19]. However, blue light therapy is not useful for medical purposes because it is difficult for blue light to affect the target area due to its low permeability caused by its short wavelength and toxic to some cells [21,22,23,24,25].

To solve these problems, this study aims to confirm the synergy of antibacterial activity through natural phytochemical extracts, previously identified to possess potent antibacterial properties [26], and visible light as a fusion therapy, such as using zinc oxide nanoparticles and essential oil together to increase the antibacterial activity effect [27]. This study assessed their absorbance and the effect of visible light on their antibacterial activity and cytotoxicity.

## 2. Materials and Methods

### 2.1. Plant Materials

A total of four species (*Caesalpinia sappan* L., *Glycyrrhiza uralensis* Fisch., *Sanguisorba officinalis* L., *Uncaria gambir* Roxb) of natural phytochemicals that showed high antibacterial activities in previous studies [26] were purchased from Samhong Medicinal Herb Market (Seoul, Republic of Korea).

### 2.2. Preparation of Plant Extracts

Preparation of plant extracts was carried out using the same method as in the previous study [26]. Plant materials were blended to powder with a grinder and extracted by shaking (110 rpm) in 70% ethanol for 24 h. The ratio of material powder to solvent was 1:10 (*w*/*v*). Supernatants were separated from crude extracts by centrifugation at 3000 rpm for 10 min and concentrated through a rotary evaporator WEV-1001V (Daihan Scientific Co., Wonju, Republic of Korea) in a vacuum at 50 °C. The concentrated extract was dissolved in 10% dimethyl sulfoxide (DMSO; Sigma Chemical Co., St. Louis, MO, USA) aqueous solution and finally filtered through a Whatman filter paper No. 2 (Whatman, Kent, UK). All samples were placed into conical tubes, sealed, and stored in a refrigerator at 4 °C until further use.

### 2.3. Light Source

An experimental device was equipped with a light-emitting diode (LED) (Jin LED, Seoul, Republic of Korea) with peak emission at 465, 520, 590, and 625 nm. Irradiation of each LED was measured by X1-5 optometer (Gigahertz-Optik, Munich, Germany). Specifications of the LED used in the experiment are shown in Table 1.

### 2.4. Bacteria Culture

*S. aureus* (ATCC 29213) and MRSA (ATCC 33591) standard strains were used in the present study [26]. These strains were purchased from the American Type Culture Collection (ATCC) (Manassas, VA, USA). MRSA isolates (CI-2 and CI-21) were originally obtained from clinical specimens and identified at Gachon University Gil Medical Center (Incheon, Republic of Korea) [28]. These isolates were preserved in a −80 °C freezer in 20% glycerol (*v*/*v*) until further use. Each strain was initially cultivated on a brain heart infusion (BHI) (Kisan Bio, Seoul, Republic of Korea) plate. A single colony was picked from each plate and pre-cultured in BHI broth at 37 °C for 24 h prior to assays. Bacterial stocks were subcultured every 3–4 weeks to maintain bacterial viability.

### 2.5. Ultra-Performance Liquid Chromatography (UPLC) Analysis

UPLC analysis was conducted under the same conditions as described in previous studies [29]. UPLC analysis of natural phytochemical extracts (3 mg/mL in 70% ethanol) was performed on an AQUITY UPLC I-Class system (Waters Corporation, Milford, MA, USA) using a BEH C18 1.7 μm column (2.1 × 100 mm) through Korea Plant Extract Bank (KPEB) (Daejeon, Republic of Korea). The sample was separated by a mobile phase consisting of distilled water with 0.1% formic acid (A) and acetonitrile with 0.1% formic acid (B). The column was set at 35 °C and the injection volume was 1 μL. The eluent was program set as follows: 0 min 92% A (8% B), 1.0 min 92% A (8% B), 16.0 min 30% A (70% B), 17.0 min 0% A (100% B), 19.0 min 0% A (100% B), 19.3 min 92% A (8% B), and 22.0 min 92% A (8% B). The flow rate was 0.4 mL/min.

High-resolution electrospray ionization mass spectrometry (HRESIMS) data were obtained on a Waters Vion QTOF/MS spectrometer in a negative/positive electrospray ionization (ESI^−^/ESI^+^) mode. MS conditions were as follows: desolvation gas (N_2_) flow rate = 800 L/h, desolvation gas temperature = 350 °C, source temperature = 110 °C, capillary voltage = 300 V, cone voltage = 40 V, and *m*/*z* range = 100–1500 Da.

### 2.6. Absorbance Measurement of Natural Phytochemical Extracts

Absorbance was measured for natural phytochemical extracts to determine the correlation between absorbance and wavelength of a specific component in the extract with a multimode plate reader (Tecan, Infinite™ M200 PRO, Männedorf, Switzerland). Absorbance was measured at 2 nm intervals from 300 nm to 800 nm.

### 2.7. Determination of Minimum Inhibitory Concentration (MIC)

Minimum inhibitory concentration (MIC) for the combination of medical plant extract and visible light was measured to determine synergistic effect on antibacterial activity against *S. aureus.* MIC was determined using the broth microdilution method according to a previous study [26] with slight modifications. Briefly, 200 μL of samples were inoculated to first columns of 96-well microplates and serially diluted two-fold (ranging from 1 to 1/4 MIC resulting from the previous study [26]). Then, 100 μL of *S. aureus* bacterial suspension (1 × 10^6^ CFU/mL) was inoculated into wells. The total volume was 200 μL per well. The microplate was irradiated with light of a specific wavelength at intervals of 10 min from 0 to 60 min and incubated in BHI broth with shaking (110 rpm) at 37 °C for 24 h. The optical density (OD) was measured at 595 nm with a spectrophotometer (Multiskan FC; Thermo Fisher Scientific, Waltham, MA, USA). MIC was defined as the lowest concentration that inhibited the visible growth of bacteria. The criteria for enhancing antibacterial activity were determined according to the fractional inhibitory concentration (FIC) value. FIC_I_ was calculated using the following equation: FIC_I_ = FIC_E_ + FIC_v_, where FIC_E_ was the MIC of the extract in combination/MIC of extract alone and FIC_v_ was the minimum inhibitory time (MIT) of visible light in combination/MIT of visible light alone. Results were interpreted as follows: synergistic interaction, FIC_I_ ≤ 0.5; partial synergy, 0.5 < FIC_I_ ≤ 0.75; additive interaction, 0.75 < FIC_I_ ≤ 1.0; indifferent, 1.0 < FIC_I_ ≤ 4.0; and antagonistic interaction, FIC_I_ > 4.0 [30].

### 2.8. Measurement of Synergy Effect of Antibacterial Activity against Methichillin-Resistant Staphylococcus aureus (MRSA)

Antibacterial activity for the combination of medical plant extract and visible light was measured to determine synergistic effect on antibacterial activity against MRSA, and MIC was determined using the broth microdilution method according to a previous study [26] with slight modifications. Briefly, 100 μL of 1/4 MIC samples in which synergistic effect on antibacterial activity was found due to visible light irradiation in Materials and Methods 2.7 were inoculated to wells of 96-well microplates. Then 100 μL of each MRSA bacterial suspension (1 × 10^6^ CFU/mL) was inoculated into wells. The total volume was 200 μL per well. The microplate was irradiated with light of a specific wavelength under the same conditions as those when enhanced antibacterial activity against *S. aureus* at 1/4 MIC was found. The optical density (OD) was measured at 595 nm with a spectrophotometer.

### 2.9. Cell Culture

Human skin keratinocytes (HaCaT; ATCC PCS-200-011) cell line was purchased from American Type Culture Collection (ATCC; Manassas, VA, USA) and human lung carcinoma (A549; KCLB 10185) cell line was purchased from Korean Cell Line Bank (KCLB, Seoul, Republic of Korea). These cells were grown in 25 cm^2^ flasks with 4 mL of Roswell Park Memorial Institute Medium (RPMI 1640; Gibco, Grand Island, NY, USA) with 10% fetal bovine serum added (FBS; Gibco) and 1% penicillin and streptomycin (Sigma Chemical Co., St. Louis, MO, USA). They were cultured at 37 °C in a CO_2_ cell chamber (MMM Group, Planegg, Germany) with 5% CO_2_. When cells fill 80–90% of the bottom of the 25 cm^2^ flask, cells were dissociated with 4 mL of trypsin-ethylenediaminetetraacetic acid (Trypsin-EDTA; Gibco) solution. After centrifugation at 500 rpm for 10 min at 4 °C, cells were re-suspended with RPMI 1640 to 2 × 10^4^ cells/mL and plated at 100 μL per well in a 96-well microplate. After incubation for 6 h at 37 °C with 5% CO_2_, cell physiological activities were performed using exponential phase cells.

### 2.10. Measurement of Cell Physiological Activity

Cell physiological activities after treatment with natural phytochemicals and visible light were analyzed using methods of measuring cell proliferation and cell metabolic activity of Rohringer et al. [31] with slight modifications. Briefly, 100 μL of a natural phytochemical extract that showed synergistic effect on antibacterial activity against MRSA strains at 1/2 MIC was inoculated so that the total volume was 200 μL. Afterwards, cell proliferation and cell metabolism were measured at 24 h intervals for 72 h at 37 °C and 5% CO_2_ after irradiation with visible light under the same conditions under which the MIC appeared when combined with 1/4 MIC of a natural phytochemical extract. Cell proliferation and toxicity were examined by removing the solution from each well, inoculating 100 μL of 0.05% Trypsin-EDTA solution, and incubating cells for 10 min at 37 °C with 5% CO_2_. The separated cell solution was mixed with an equal amount of trypan blue solution (Sigma Chemical Co., St. Louis, MO, USA). After reacting for 5 min at room temperature, the number of viable cells was measured using a hemocytometer. Cytotoxicity was judged to be toxic if the cell viability of the experimental group was less than 70% when comparing the experimental group and the control group. For cell metabolism, 20 μL of 5 mg/mL MTT solution was inoculated per well and reacted for 2 h. After the reaction, the plate was washed with phosphate-buffered saline (PBS; Gibco), inoculated with 200 μL of DMSO, and reacted at room temperature for 15 min. Afterwards, cellular metabolism was determined by measuring absorbance at 595 nm via spectrophotometry.

### 2.11. Statistical Analysis

Statistical analysis was performed by Microsoft Excel 2016 (Microsoft, Redmond, WA, USA) and SigmaPlot version 12.0 (Systat Software, Palo Alto, CA, USA). Average values were calculated as means and standard deviation (±SD). Statistical differences were assessed by analysis of variance (ANOVA). Results were considered statistically significant if the *p*-value was less than 0.05. All experiments were performed in duplicate three times.

## 3. Results and Discussion

### 3.1. Ultra-Performance Liquid Chromatography (UPLC) Analysis

UPLC analysis for each extract and mass spectrum (MS) and UV spectrum for a specific peak were conducted through the Korea Plant Extract Bank (Table 2). In the case of analysis for specific peaks, the structure was predicted through comparative analysis with the existing literature for each natural phytochemical based on results of the MS and UV spectra. As a result of the analysis, for all natural phytochemical extracts, peaks in various areas, not a single peak, appeared in all analysis results. When comparing these results with the existing literature on each natural phytochemical, the main expected effective phytochemicals of each natural phytochemical extract were episappanol; protosappanin C; brazilin; a mixture of protosappanin B, isoprotosappanin B, and sappanol for CS at 280 nm [32]; liquiritin and glycyrrhizin for GU at 254 nm [33]; quercetin and proanthocyanidins for SO at 360 nm [34]; and catechin for UG at 280 nm [35] because of the UPLC in the same UV area. Some of the above components are also expected to create synergy with visible light.

### 3.2. Absorbance Measurement of Natural Phytochemical Extracts

**Figure 1:**
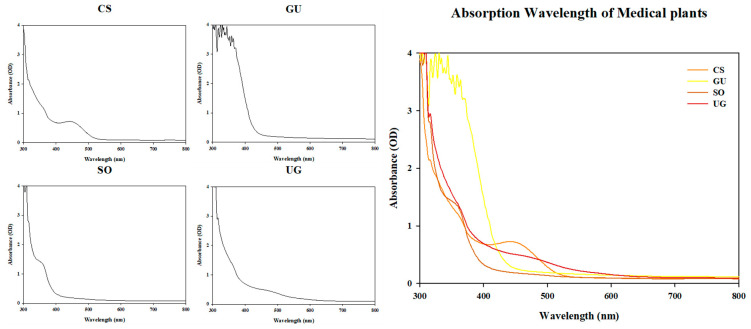
Absorption peaks of natural phytochemical extracts used in the experiment. The absorbance of each natural phytochemical extract at 4 mg/mL was measured. CS is *Caesalpinia sappan* L.; GU is *Glycyrrhiza uralensis* Fisch.; SO is *Sanguisorba officinalis* L.; UG is *Uncaria gambir* Roxb.

The absorption peak of each natural phytochemical extract was measured at 2 nm intervals from 300 nm to 800 nm (Figure 1). CS and SO showed absorption peaks at 440 and 350 nm, respectively. However, GU and UG did not show specific peaks. If the synergistic effect on the antibacterial activity of the natural phytochemicals through photoactivity is correlated with the absorbance peak, it is expected that the synergistic effect on the antibacterial activity of CS will be the highest when irradiated with blue light (465 nm) close to the 440 nm wavelength. However, as a result of measuring the antibacterial activity, CS, which showed an absorption peak at 440 nm with blue light, did not show a synergistic effect on antibacterial activity. Only SO and UG, which did not show absorption peaks, showed a synergistic effect on antibacterial activity at a concentration of one-fourth MIC. It was confirmed that the absorbance peak of the crude extract was not associated with photoactivation.

### 3.3. Determination of Minimum Inhibitory Concentration (MIC)

**Figure 2:**
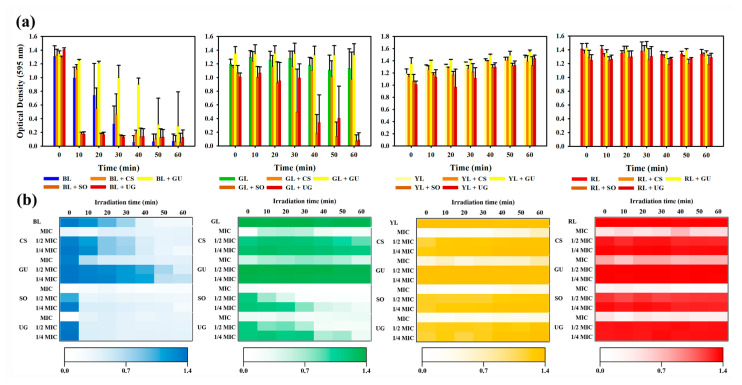
Synergistic effect on natural phytochemical extracts on antibacterial activity. (**a**) One-fourth MIC according to the wavelength of visible light. Antibacterial activity was confirmed by measuring absorbance at 595 nm. BL is 465 nm light, GL is 520 nm light, YL is 590 nm light, and RL is 625 nm light. (**b**) Heat map of enhancement of antibacterial activity according to the concentration of natural phytochemical extract and irradiation time based on visible light wavelength. Antibacterial activity was confirmed by measuring absorbance at 595 nm.

The MIC of each natural phytochemical extract at each wavelength for *S. aureus* was determined by measuring the absorbance at 595 nm using a spectrophotometer (Figure 2a). When BL was irradiated, the minimum inhibition of *S. aureus* was measured from 40 min. When one-fourth MIC of the natural phytochemical extract and BL were simultaneously treated, the minimum inhibition of *S. aureus* was measured from 10 min for SO, 10 min for UG, 40 min for CS, and 50 min for GU. When irradiated with GL, the minimum inhibition of *S. aureus* was not measured up to 60 min when GL was single-treated. When one-fourth MIC of the natural phytochemical extract and GL were simultaneously treated, the minimum inhibition of *S. aureus* was measured from 40 min for SO and 60 min for UG. However, the minimum inhibition was not measured for CS and GU up to 60 min. When irradiated with YL and RL, the minimum inhibition of *S. aureus* was not measured for up to 60 min with either single treatment of light of each wavelength or simultaneously with natural phytochemical extracts of one-fourth MIC. The synergistic effect on antibacterial activity was determined based on the FIC_I_ according to the FIC_V_. When results were analyzed, CS and GU at one-fourth MIC were not found to have any synergistic effects on antibacterial activity at any wavelength. However, SO and UG were found to show synergistic effects on antibacterial activity with BL and GL. For SO and UG at one-fourth MIC, the minimum inhibition time with BL showed a strong synergy because FIC_I_ ≤ 0.5 was 10 min, which was equivalent to one-fourth of the BL single processing result at 40 min. In addition, the minimum inhibition time of GL single treatment was not measured up to 60 min, although there was a possibility of synergy because the minimum inhibition time was 40 min for one-fourth MIC SO and 60 min for one-fourth MIC UG, both of which fell in the range of 0.25 ≤ FIC_I_ < 1.25.

To determine the synergy conditions for antibacterial activity, a heat map was prepared according to the concentration of the natural phytochemical extracts and the irradiation time of visible light (Figure 2b). Natural phytochemical extracts were diluted two-fold from the MIC and measured for *S. aureus* treated with a single extract at one-fourth MIC. Visible light irradiation time was measured from 0 min to 60 min at intervals of 10 min. When BL was used for irradiation and measurement of antibacterial activity was performed at intervals of 10 min, measurement time results of antibacterial activity for half MIC and one-fourth MIC of natural phytochemical extracts were the same. When GL was used for irradiation and when GL single treatment and CS or GU were simultaneously used for treatment, antibacterial activity was not measured up to 60 min. During the simultaneous treatment with GL or SO, the half MIC of SO showed an antibacterial activity from 20 min and the one-fourth MIC of SO showed an antibacterial activity from 40 min. When GL single treatment and CS or GU were simultaneously used for treatment, the antibacterial activity was not measured up to 60 min. During simultaneous treatment with GL or UG, the antibacterial activity was measured from 30 min at half MIC and from 60 min at one-fourth MIC. When irradiated with YL or RL, the antibacterial activity was not measured in any situations up to 60 min.

When comparing single treatment with each of four visible light wavelengths, the shorter the irradiation wavelength and the longer the irradiation time, the higher the antibacterial activity. These results confirmed that antibacterial active ingredients included in the natural phytochemical extract used in this experiment showed a concentration-dependent antibacterial activity. In addition, when the concentration of natural phytochemical extract was the same, antibacterial activity of the natural phytochemical extract had the following trend: BL > GL > YL = RL. Thus, the four natural phytochemical extracts used in this experiment are expected to show synergy in antibacterial activity by specific wavelengths rather than the absorption peaks of the extracts.

### 3.4. Measurement of Synergy Effect of Antibacterial Activity against Methichillin-Resistant Staphylococcus aureus (MRSA)

**Figure 3:**
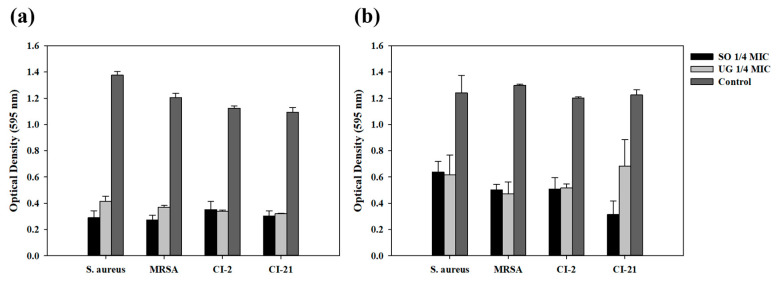
Synergistic effect of antibacterial activity against *S. aureus* and MRSA. (**a**) Optical density against *S. aureus* and MRSA when simultaneously treated with 1/4 MIC of natural phytochemical extract and irradiated at 465 nm for 10 min. (**b**) Optical density against *S. aureus* and MRSA when simultaneously treated with 1/4 MIC of natural phytochemical extract and irradiated at 520 nm for 40 min.

Antibacterial activity against MRSA was measured to determine whether the synergistic effect on antibacterial activity works regardless of antibiotic resistance. Antibacterial activity was measured using one MRSA standard strain and two clinically isolated MRSA strains used in a previous study [26] under the same conditions as those in which synergistic effect on antibacterial activity was shown at one-fourth MIC of SO or UG for the *S. aureus* standard strain (Figure 3). Two types of conditions that show synergistic effects on antibacterial activity were targeted: one-fourth MIC of SO or UG with irradiation at 465 nm for 10 min and at 520 nm for 40 min. As a result, under both conditions, all MRSA strains and *S. aureus* showed similar results. Synergistic effect on antibacterial activity compared to control was observed. This results showed that the synergistic effect between visible light and natural phytochemicals used in this study on antibacterial activity occurred regardless of antibiotic resistance.

### 3.5. Measurement of Cell Physiological Activity

**Figure 4:**
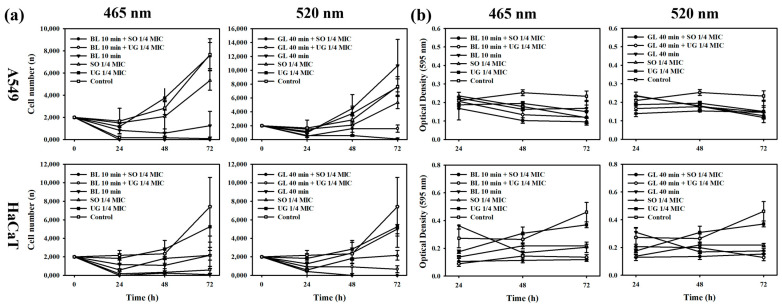
Cell physiological activities when simultaneously treated with 1/4 MIC of natural phytochemical extract and irradiated at 465 nm for 10 min against A549 and HaCaT cell lines. (**a**) Cell proliferation. (**b**) Cellular metabolic activity.

When cell proliferation was determined under conditions where antibacterial activity synergy occurred, both A549 cells and HaCaT cells showed a survival rate of less than 10%, indicating a strong cytotoxicity. When single irradiation at each wavelength was performed, 465 nm showed toxicity to both cells, with a survival rate of 16% for A549 cells and 29% for HaCaT cells. However, irradiation at 520 nm resulted in a high proliferation rate of 139% for A549 cells. Although HaCaT cells showed toxicity at 67%, they showed a relatively high survival rate. In the case where light was not irradiated, A549 cells showed a survival rate of 70% upon treatment with one-fourth MIC of SO and 98% upon treatment with one-fourth MIC of UG, indicating no toxicity. HaCaT cells showed a survival rate of 29% upon treatment with one-fourth MIC of SO. However, they showed a survival rate of 71% upon treatment with one-fourth MIC of UG (Figure 4a).

For cellular metabolic activity, A549 cells showed a decrease in absorbance compared to controls under all conditions. The metabolic activity decreased over time at 465 nm. Metabolic activity of HaCaT cells increased over time in control or single treatment group with UG one-fourth MIC, but decreased for the remaining conditions at 465 nm. Both A549 cells and HaCaT cells at 520 nm showed results similar to those at 465 nm (Figure 4b).

These results indicate that the combination therapy of the natural phytochemicals contained in the natural phytochemical extract used in this study with visible light showing synergistic effect on antibacterial activity was not specifically toxic to bacteria. Therefore, it is expected to be a mechanism that caused non-specific damage by increasing ROS production. Through this, the possibility of photodynamic utilization in the visible light region with a long wavelength was confirmed, as using 520 nm compared to 465 nm could reduce damage to areas other than the affected area when using combination therapy of natural phytochemicals and visible light.

## 4. Conclusions

In this study, we confirmed the synergy of antibacterial activity of four natural phytochemical extracts and visible light against MRSA. As a result, SO and UG showed synergistic effect on antibacterial activity at 465 nm and 520 nm. Antibacterial activity was confirmed when both SO and UG were irradiated for 10 min at 465 nm and when SO was irradiated for 40 min and UG was irradiated for 60 min at 520 nm. Antibacterial activity correlation between the absorption peak of the crude extract and the visible light wavelength seemed to be small, and synergistic effect on antibacterial activity was confirmed to increase in proportion to the light irradiation time and inversely proportional to the length of the wavelength. Also, the antibacterial activity of SO and UG by visible light was confirmed to be active regardless of antibiotic resistance. It was confirmed that the antibacterial activity mechanism causes non-specific damage regardless of the cell and bacteria, and it was confirmed that 520 nm causes less damage to cells than 465 nm under conditions where the one-fourth MICs of SO and UG show antibacterial activity. These results confirmed that certain plant extracts can be used as a photosensitizer through visible light. Additionally, it was shown that more efficient antibacterial activity against MRSA was possible when using the fusion therapy compared to using the plant extract and photodynamic therapy alone.

Currently, blue light is mainly used in the case of antibacterial activity in the visible light area using natural phytochemicals. Blue light exhibits antibacterial activity by activating bacterial protoporphyrin lX (PplX) to increase ROS. Various studies are being conducted on blue light because it is less cytotoxic than ultraviolet rays. Some studies show that it can be specifically toxic when used with natural phytochemicals such as carvacrol [7,18,19,20]. However, the low human tissue permeability due to the short wavelength of blue light is underutilized [22,25,36,37]. Blue light can damage not only bacteria but also host cells [21,38,39]. This study showed that certain natural phytochemicals could be active according to specific wavelengths or irradiation time, not because they were active at absorption peaks among the four wavelengths used in the experiment.

Therefore, if the light of a longer wavelength than blue light is used in treatment in combination with a specific natural phytochemical, it will solve the problem related to short human tissue permeability and damage to host cells, which are disadvantages of a conventional blue light therapy. In addition, red light and green light can affect cell proliferation and regeneration without significantly affecting cell metabolic activity when a single treatment is performed on cells [31]. It is thought that natural phytochemicals active at various wavelengths can have various advantages in treating human tissue lesions if used in photodynamic treatment. However, since the experiment time was limited to one hour in this study, whether there was antibacterial activity during a single treatment of a wavelength other than BL and whether the antibacterial activity was enhanced when sufficient time was given at wavelengths longer than GL was unclear. Therefore, in future studies, it will be necessary to prove that natural phytochemicals do not increase antibacterial activity at a specific wavelength. Also, because this study was conducted using only crude extract, there is a lack of analysis of ingredients that can be used as a photosensitizer. To solve this problem, in future research, it is necessary to confirm the antibacterial activity of each fraction according to the characteristics of the ingredients and analyze the ingredients using methods such as total phenol content and total flavonoid content and analysis of additional antibacterial activity mechanisms through methods such as total ROS detection is also necessary. In addition, if visible light, which is effective in cell physiological activity, and natural phytochemical extracts, which show antibacterial activity enhancement through photoactivation, can be combined to confirm that both antibacterial and cell physiological activities are enhanced, it will increase the utilization of photodynamic therapy in the treatment of human tissue lesions.

## Figures and Tables

**Table 1:** LED specifications used in this study.

Wavelength (nm)	Voltage (V)	Current (mA)	Irradiance (mW/cm^2^)	Distance Between Light Source and Solution Surface (mm)
620–625	2–2.2	20	73.29	6
591–593	2–2.2	20	84.17	6
520–522.5	3–3.2	20	71.68	6
465–467.5	3–3.2	20	93.34	6

**Table 2:** Expected effective phytochemical analysis of each natural phytochemical extract [29].

Extracts	RT (min)	Molecular Formula	Molecular Weight (*m*/*z*)	Phytochemical Name
CS	2.98	C_16_H_14_O_5_	285.07	Brazilin
	3.10	C_16_H_16_O_6_	304.30	Protosappanin B
GU	4.35	C_26_H_30_O_13_	550.51	Liquiritin apioside
	9.48	C_42_H_62_O_16_	822.94	Glycyrrhizin
SO	6.60	C_16_H_10_O_11_S	410.31	2,7-*O*-methyl-8-(sulfooxy)ellagic acid
UG	3.07	C_15_H_14_O_6_	290.27	Catechin

CS: *Caesalpinia sappan* L.; GU: *Glycyrrhiza uralensis* Fisch.; SO: *Sanguisorba officinalis* L.; UG: *Uncaria gambir* Roxb.; RT: Retention time.

## Data Availability

Data are contained within the article.
