# Correction: Jeong, J.-Y.; Hwang, Y.-J. Natural Phytochemical and Visible Light at Different Wavelengths Show Synergistic Antibacterial Activity against *Staphylococcus aureus*. *Pharmaceutics* 2024, *16*, 612

**DOI:** 10.3390/pharmaceutics16111478

**Published:** 2024-11-20

**Authors:** Jae-Young Jeong, You-Jin Hwang

**Affiliations:** 1 Department of Biohealth & Medical Engineering, College of IT Convergence, Gachon University, Seongnam 13120, Republic of Korea; jini656565@gachon.ac.kr; 2 Department of Biomedical Engineering, College of IT Convergence, Gachon University, Seongnam 13120, Republic of Korea

In the original publication [1], there was a mistake in “Table 1. LED specifications used in this study”, as published. The irradiance of the light source was measured by increasing the current for each LED for each wavelength, a graph of irradiance against current was created based on the measured values, and the irradiance used in this study was derived by substituting the used current value into the created graph. However, because the equipment that measured irradiance in this study was for measuring the irradiance of surface light sources, the measured irradiance of point light sources was too high. Therefore, after measuring the irradiance at each angle of the LED used in the experiment, we would like to modify the original values by substituting the values into the equation to obtain the average value according to the area of irradiance used. The corrected Table 1 appears below.

There was an text error in the original publication, Sections 2.9, 3.3 and 4.

Referring to the last sentence of “2.9. Cell Culture”, it is correct to proceed according to this method when subculturing. However, in the later method of measuring cell physiological activity, an amount of 2 × 10^3^ cells/well was used, but since this information was omitted, we plan to include this information in the cell culture method. Therefore, it has now been corrected as follows.

*2.9. Cell Culture*

After centrifugation at 500 rpm for 10 min at 4 °C, cells were re-suspended with RPMI 1640 to 2 × 10^4^ cells/mL and plated at 100 μL per well in a 96-well microplate. After incubation for 6 h at 37 °C with 5% CO_2_, cell physiological activities were performed using exponential phase cells.

Due to the results were reinterpreted by re-measuring the irradiances of the LED, antibacterial activity synergy was found only at 465 nm and 520 nm even though irradiance was similar, so the contents were revised in the last sentence of “3.3. Determination of Minimum Inhibitory Concentration (MIC)” and part of the second paragraph of “4. Conclusions” as follows:

*3.3. Determination of Minimum Inhibitory Concentration (MIC)*

Thus, the four natural phytochemical extracts used in this experiment are expected to show synergy in antibacterial activity by specific wavelengths rather than the absorption peaks of the extracts.

**4. Conclusions**

This study showed that certain natural phytochemicals could be active according to specific wavelengths or irradiation time, not because they were active at absorption peaks among the four wavelengths used in the experiment.

The authors state that the scientific conclusions are unaffected. This correction was approved by the Academic Editor. The original publication has also been updated.

## Figures and Tables

**Table 1:** LED specifications used in this study.

Wavelength (nm)	Voltage (V)	Current (mA)	Irradiance (mW/cm^2^)	Distance Between Light Source and Solution Surface (mm)
620–625	2–2.2	20	73.29	6
591–593	2–2.2	20	84.17	6
520–522.5	3–3.2	20	71.68	6
465–467.5	3–3.2	20	93.34	6
